# An investigation on NO removal by wet scrubbing using NaClO_2_ seawater solution

**DOI:** 10.1186/s40064-016-2528-3

**Published:** 2016-06-17

**Authors:** Zhitao Han, Shaolong Yang, Dekang Zheng, Xinxiang Pan, Zhijun Yan

**Affiliations:** Marine Engineering College, Dalian Maritime University, No.1, Linghai Road, Dalian City, 116026 Liaoning Province People’s Republic of China

**Keywords:** Nitric oxide, Scrubbing, Oxidation, Sodium chlorite, Seawater

## Abstract

The experiments were conducted to investigate the NO removal by wet scrubbing using NaClO_2_ seawater solution in a cyclic scrubbing mode. Results show that, when the concentration of NaClO_2_ in scrubbing solution is higher than 10 mM, a complete removal of NO can be achieved during the cyclic scrubbing process. The breakthrough time for seawater with 15 mM NaClO_2_ is enhanced by 34.3 % compared with that for NaClO_2_ freshwater. The extension of the breakthrough time for NaClO_2_ seawater is mainly ascribed to the improved utilization of NaClO_2_ in the solution. The good buffering ability of seawater could suppress the acidic decomposition of NaClO_2_ into ClO_2_ effectively. The analysis of reaction products indicates that the main anions in the spent liquor are chloride ions and nitrate ions. The calculation of NaClO_2_ utilization according to the ion chromatography also agrees well with the experimental results of breakthrough times.

## Background

Air pollution is currently one of the major problems worldwide, resulting that more rigorous environmental laws are introduced. The main source of air pollution is the combustion process of fossil fuels used in power plants, vehicles, ships and other incineration processes (Skalska et al. 2010). Sulfur oxides (principally SO_2_), nitrogen oxides (NO_x_), particulate matter (PM) are the key combustion-generated air contaminants. Since NO_x_ have been implicated in a variety of environmental effects such as acid rain, photochemical smog, tropospheric ozone layer depletion and even global warming, they are considered as the primary pollutants of the atmosphere (Wei et al. 2009a).

At present, the implementation of stringent regulations of NO_x_ emission requires the development of new technologies and improvement of currently used methods for NO_x_ removal from exhaust gas. Effective reduction of NO_x_ emissions from both stationary and mobile sources poses a major challenge. Technologies for the NO_x_ removal can be divided into combustion control and post-combustion treatment. Combustion control aims at reducing the formation of NO_x_ during the combustion of fossil fuel (Adewuyi et al. 1999). Post-combustion control methods include a variety of techniques such as selective catalytic reduction (SCR) (Zhang et al. 2015; Jiang et al. 2015), selective non-catalytic reduction (SNCR) (Lee et al. 2008), thermal DeNOx and scrubbing. SCR can remove NO_x_ with an efficiency of 80–95 % and it has been applied in power plants extensively. Up to date, SCR is also considered as one of the most promising techniques for ocean-going ships. But it requires additional space, high investment and operating cost. Another major concern of SCR is the deleterious effect of SO_2_-laden flue gas on the life of catalyst which is an important factor of this technology’s cost. SNCR approach requires high reaction temperature (900–1000 °C) with an elaborate temperature control to avoid ammonia slip and to achieve effective NO_x_ removal. Scrubbing method is one of the advanced air pollution control technologies. It has the ability of removing other acid gases and particulates simultaneously (Makansi 1990).

Nitric oxide (NO) is a major component of NO_x_ emitted from incineration processes and is of low solubility in aqueous solution, so it can not be removed easily from the flue gas by scrubbing. But a feasible method is to oxidize NO to other soluble NO_x_ species (NO_2_, N_2_O_3_, N_2_O_4_, N_2_O_5_, etc.). For this purpose, various oxidants such as hydrogen peroxide (H_2_O_2_) (Liu and Zhang 2011), potassium permanganate (KMnO_4_) (Chu et al. 2001a), peracids (Littlejohn and Chang 1990), ferrous-chelating agents (Wang et al. 2007; Yan et al. 2015), sodium hypochlorite (NaClO) (Chen et al. 2005) and sodium chlorite (NaClO_2_) (Deshwal et al. 2008; Wei et al. 2009b) have been investigated to enhance the NO removal efficiency of the scrubbing solution. Among these oxidants, NaClO_2_ has been found one of the most promising chemicals for NO oxidation. In the late 1970s, Sada et al. performed early studies on the absorption of NO_x_ in a NaClO_2_ solution and proposed the overall reactions of NO and NO_2_ with NaClO_2_ in alkaline solutions (Sada et al. 1978, 1979). Brogren et al. studied the kinetics of the absorption of NO in an alkaline solution of NaClO_2_ and found that the chlorite ion oxidized NO mainly to NO_2_ or *NO*_2_^−^ and NO_2_ to *NO*_3_^−^ (Brogren et al. 1998). Yang and co-workers conducted experiments with acid solution of NaClO_2_ and determined the overall reaction of NO with NaClO_2_ in acidic conditions (Yang and Shaw 1998). Apart from the considerable studies on kinetics of NO absorption with NaClO_2_ (Hsu et al. 1998; Chu et al. 2001b), more and more research on simultaneously removal of NO_x_ and other pollutants (such as SO_2_ and Hg) by scrubbing NaClO_2_ solution has been conducted throughout the world (Jin et al. 2006; Huston et al. 2008; Zhao et al. 2010; Park et al. 2015). However, all of the work above-mentioned is focused on experimentations using NaClO_2_ freshwater solution. Since seawater has some unique properties such as the natural alkalinity (pH 7.5–8.3) and good buffering ability, it has already been used to remove SO_2_ from the flue gas of on-going ships and onshore power plants (Andreasen et al. 2007; Vidal et al. 2007; Lan et al. 2012). To the best of our knowledge, research on NO removal by using NaClO_2_ seawater has not been reported yet. The purpose of this work is to use seawater to take place of fresh water as solvent and to study the effect of seawater on the NO removal of NaClO_2_ solution. The experimental results showed that NaClO_2_ seawater can remove NO from the simulated flue gas with a high efficiency. Besides, the breakthrough time for NO absorption with NaClO_2_ seawater is obviously enhanced due to the buffering ability of seawater compared with that for NaClO_2_ freshwater and the possible reaction mechanism has also been discussed.

## Experimental

### Materials

The reagents used in this investigation were: N_2_ pure gas, NO span gas (10 %) with N_2_ as the balance gas, NaClO_2_, sodium chloride (NaCl), magnesium chloride hexahydrate (MgCl_2_∙6H_2_O), sodium sulfate (Na_2_SO_4_), anhydrous calcium chloride (CaCl_2_), potassium chloride (KCl), sodium bicarbonate (NaHCO_3_), sodium hydroxide (NaOH) and hydrochloric acid (HCl). All chemicals were analytical reagent (AR) degree without further purification. The deionized water was prepared in a two-stage ELGA PURELAB Option R15 purification system and had a resistivity of 15 MΩ∙cm.

The substitute ocean water, which is prepared according to an American national standard (ASTM 2008), is used as seawater solvent in the experiments. For simplicity, minor elements, occurring naturally in concentration below 0.2 g/L, are not included. The process for preparing the substitute ocean water is as follows: firstly, stock solution No. 1 is prepared by dissolving 11.11 g MgCl_2_∙6H_2_O and 1.16 g CaCl_2_ in 50 mL deionized water. Secondly, stock solution No. 2 is prepared by dissolving 0.695 g KCl and 0.201 g NaHCO_3_ in 50 mL deionized water. Thirdly, dissolve 24.53 g NaCl and 4.09 g Na_2_SO_4_ in 900 mL deionized water and then add stock solution No. 1 and No. 2 slowly with vigorous stirring. Finally, adjust the pH value of the synthetic seawater to 8.2 with 0.2 M NaOH solution.

The scrubbing solution of various NaClO_2_ concentrations (5, 10, 15 and 20 mM) is prepared by adding certain amount of NaClO_2_ into deionized water and seawater, respectively. The initial pH values of NaClO_2_ solution are listed in Table 1. In order to investigate the buffer capacity of NaClO_2_ solution on the NO removal, 20 mM NaClO_2_ seawater and freshwater solution are titrated with 0.2 M HCl, respectively.

**Table 1:** The initial pH values of the scrubbing solution of various NaClO_2_ concentrations

NaClO_2_ concentration (mM)	The pH value of freshwater solution	The pH value of seawater solution
0	7.09	8.20
5	9.62	8.11
10	9.88	8.41
15	10.1	8.42
20	10.5	8.61

### Experimental setup

**Fig. 1:**
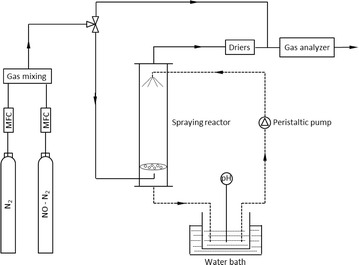
Schematic diagram of the gas scrubbing system

A schematic diagram of the experimental system is shown in Fig. 1. This system consists of a simulated flue gas supply unit, a countercurrent spraying reactor and a gas analyzing system. N_2_ pure gas and NO span gas are provided from separate air bottles and metered through mass flow controllers (MFC). The synthetic flue gas is obtained from the feed gases by blending with an online mixer and then it is introduced into the countercurrent spraying column made of polymeric methyl methacrylate (PMMA). A spraying nozzle (B1/4TT-SS+TG-SS0.4, Spraying System Co.) is mounted at the top of the column. The size of liquid droplet sprayed from the nozzle is in the range of 80–100 μm. The height and inner diameter of the column are 25 and 5 cm, respectively. The simulated flue gas continuously flows through the column and the calculated residence time of flue gas in the column is about 19.6 s. After contacting with the flue gas in counter flow, the scrubbing liquid is collected under the column in a beaker, from which the absorption liquid is pumped to the top of the column using a peristaltic pump. The total amount of the cyclic scrubbing solution is 0.2 L. The rotating speed of the peristaltic pump is set at 200 rpm, resulting in that the flow rate of the scrubbing solution is about 0.35 L/min. The scrubbing solution is kept at 20 °C by immersing the beaker in a water bath.

The scrubber gaseous effluent is analyzed with a Madur GA-21 gas analyzer which is a multi-functional flue gas analyzer. Electrochemical sensors are used for the measurement of gas concentration. NO and NO_2_ are measured directly using the electrochemical cells while the NO_x_ concentration is calculated by the analyzer as a simple sum of measured NO and NO_2_ concentration. In order to protect the flue gas analyzer, the simulated flue gas is dried by anhydrous CaCl_2_ particles after being scrubbed. A pH meter is used to on-line monitoring the pH value and temperature of the scrubbing solution. After scrubbing, sample solution is withdrawn from the spent solution in the beaker for ion chromatographic analysis (Dionex ICS-1500).

## Results and discussion

**Fig. 2:**
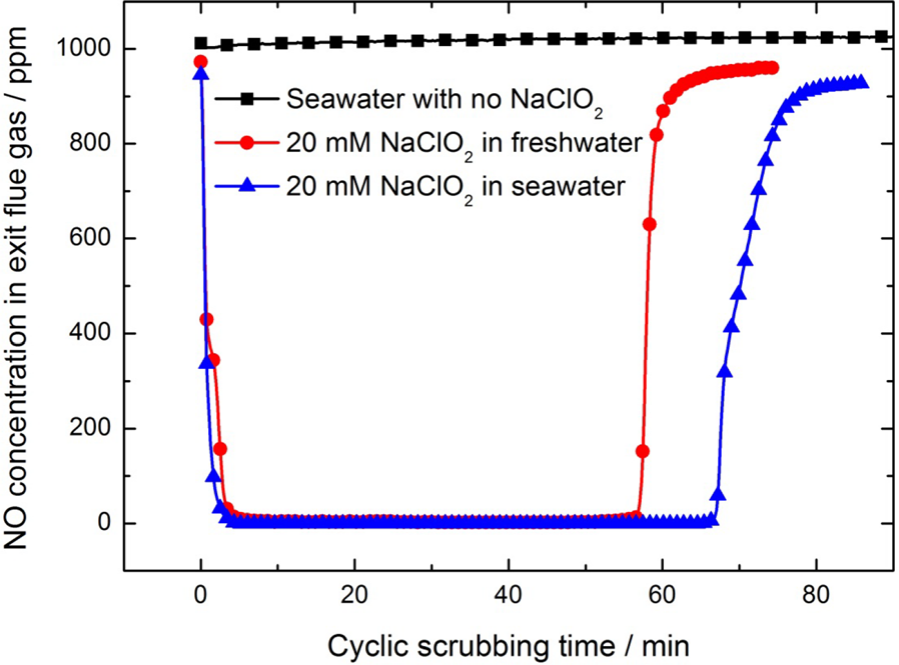
NO absorption of 20 mM NaClO_2_ scrubbing solution

The experiments of cyclic scrubbing the flue gas continuously by using different solutions to remove NO had been conducted. Figure 2 shows the changes of NO concentrations in exit flue gas during the scrubbing process. From the results, we can see that when seawater without NaClO_2_ addition is used to treat the flue gas, NO concentration in exit flue gas nearly does not change, which demonstrates that seawater itself does not react with NO. For both freshwater and seawater, when there is 20 mM NaClO_2_ added in the solution, NO concentration decreases sharply to zero within several minutes at the beginning of the scrubbing process. It suggests that NO in flue gas has been completely removed. With the proceeding of the cyclic scrubbing process, the complete removal of NO can last for tens of minutes. Finally, NO concentration increases fast to the initial level (about 1000 ppm), which indicates that NaClO_2_ in the solution has been depleted.

**Fig. 3:**
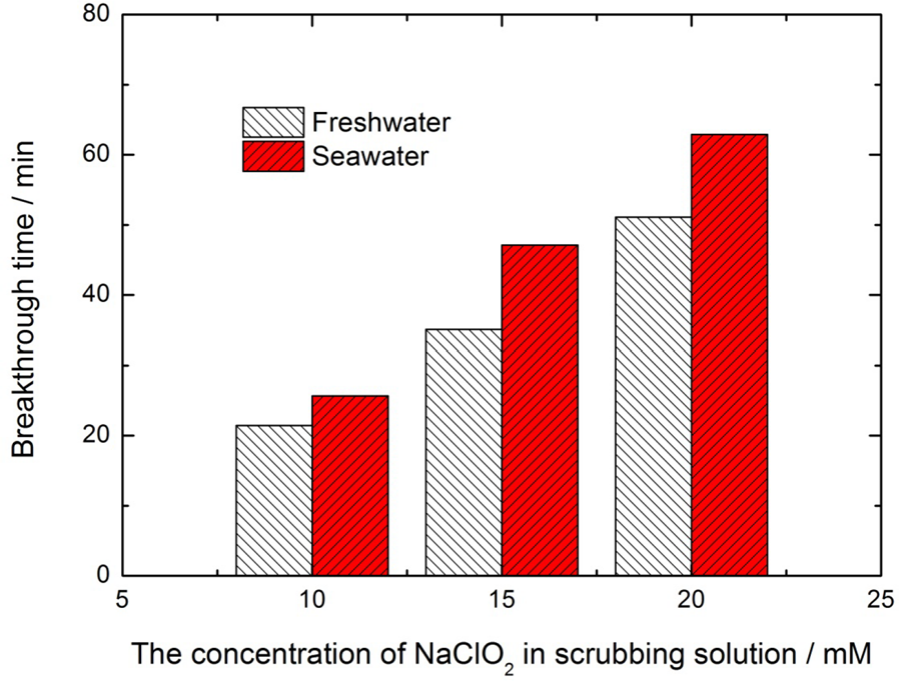
The breakthrough times for NO absorption for cyclic scrubbing of NaClO_2_ solutions

From Fig. 2, it can be seen that, with the same concentration of NaClO_2_ in the solution, the breakthrough times, which refers to the duration of complete removal of NO from the flue gas, are obviously different from freshwater to seawater. The breakthrough times for NO absorption by scrubbing solution containing various concentrations (5–20 mM) of NaClO_2_ are illustrated in Fig. 3.

When the concentration of NaClO_2_ in the scrubbing solution is 5 mM, a complete removal of NO from the flue gas can not be achieved during the whole scrubbing process, so the breakthrough time is zero for 5 mM NaClO_2_ solution. From Fig. 3, one can see that, the breakthrough times for NaClO_2_ seawater solution have been enhanced obviously compared with those for freshwater solution of the same concentration of NaClO_2_ oxidant. When the concentration of NaClO_2_ is 15 mM, the breakthrough time for seawater can be improved by 34.3 % compared with that for freshwater. Since the breakthrough time for NO absorption during the cyclic scrubbing process represents the ability of NO absorption for the scrubbing solution, NaClO_2_ seawater exhibits much higher NO absorption potential than NaClO_2_ freshwater.

**Fig. 4:**
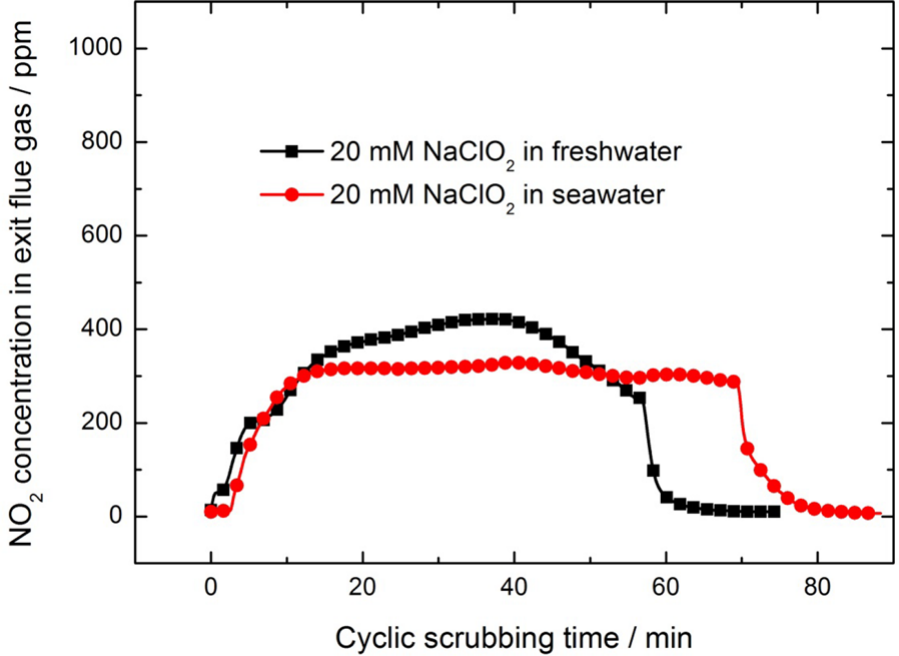
The change in concentrations of NO_2_ in exit flue gas during the scrubbing process

Figure 4 shows the change of NO_2_ concentration in exit flue gas during the cyclic scrubbing process. As the simulated flue gas treated in our experiments is the mixture of N_2_ and NO, the initial concentration of NO_2_ in exit flue gas is zero. With the start of the scrubbing process, the concentration of NO_2_ in exit flue gas increases gradually due to the oxidation of NO into NO_2_ by NaClO_2_ (Brogren et al. 1998).

1 $$2NO\;{ + }\,NaClO_{ 2} \to 2NO_{ 2} { + }\,NaCl$$

The equilibrium reaction between NO and NO_2_ will occur simultaneously as in Eqs. (2) and (3).

2 $$NO\;{ + } \,NO_{ 2} \to N_{ 2} O_{3}$$

3 $$2NO_{ 2} \to N_{ 2} O_{ 4}$$

It follows that some NO_x_ are absorbed via the hydrolysis of N_2_O_3_ and N_2_O_4_ as in Eqs. (4) and (5).

4 $$N_{ 2} O_{ 3} \,{ + }\, H_{ 2} O \to 2HNO_{ 2}$$

5 $$N_{ 2} O_{ 4}\, { + }\,H_{ 2} O \to HNO_{ 2}\, { + }\,HNO_{ 3}$$

Both N_2_O_3_ and N_2_O_4_ are very easy to react with H_2_O, so most of them will be removed during the wet scrubbing process. To some extent, N_2_O_3_ and N_2_O_4_ can be considered as intermediate products. The NO_x_ in exit flue gas are mainly NO and/or NO_2_. The reaction between NO and NO_2_, as well as the hydrolysis of N_2_O_3_ and N_2_O_4_, will not affect the analysis result of NO_x_ absorption obviously.

Figure 4 also shows that, for 20 mM NaClO_2_ seawater solution, the concentration of NO_2_ in exit flue gas is more stable than that of freshwater solution, suggesting that seawater is better for balancing the NO absorption during the cyclic scrubbing process. Besides, for 20 mM NaClO_2_ seawater solution, the total removal efficiency of NO_x_ (here it is the sum of NO and NO_2_) can be approximately calculated as 70 % during the cyclic scrubbing process.

**Fig. 5:**
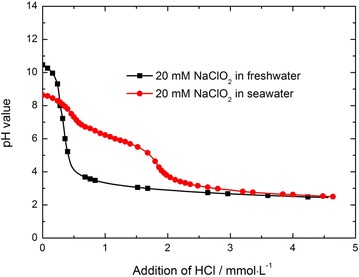
The change of pH of NaClO_2_ (20 mM) scrubbing solution with addition of HCl (0.2 M)

Compared with freshwater, artificial seawater used in our experiments contains 419.74 mM NaCl, 54.62 mM MgCl_2_, 28.79 mM Na_2_SO_4_, 10.45 mM CaCl_2_, 9.32 mM KCl and 2.39 mM NaHCO_3_. Apart from these six kinds of salts, there is a little NaOH used to adjust the mixture solution pH to 8.2. Here it can be seen that, although the components of seawater solution seem to be a little complex, it can be considered as a buffering solution, because it does not react with NO directly. In order to investigate the effect of the buffer capacity of seawater on NO absorption by NaClO_2_ solution, the experiments of titrating 20 mM NaClO_2_ solutions with 0.2 M HCl are performed. The changes in solution pH value with the addition of HCl are shown in Fig. 5.

It is known that water itself is a buffering medium, even in the absence of an added buffering reagent. Unlike water, seawater is of natural alkalinity, defined as the sum of the concentrations of the alkaline species contained in seawater. The value of alkalinity is not fixed, but changes according to the geographical area. In a general way, seawater alkalinity can be expressed as follows (Giuseppe et al. 2012):

6 $$\begin{aligned} A_{T} = \left[ {HCO_{3}^{ - } } \right] + 2\left[ {CO_{3}^{2 - } } \right] + \left[ {B\left( {OH} \right)_{4}^{ - } } \right] + \left[ {OH^{ - } } \right] + \left[ {HPO_{4}^{2 - } } \right] + 2\left[ {PO_{4}^{3 - } } \right] + \left[ {SiO\left( {OH} \right)_{3}^{ - } } \right] \hfill \\ \begin{array}{*{20}c} {\begin{array}{*{20}c} {} & {} \\ \end{array} } & { + \left[ {HS^{ - } } \right] - \left[ {H^{ + } } \right]} \\ \end{array} - \left[ {HSO_{4}^{ - } } \right] - \left[ {HF} \right] - \left[ {H_{3} PO_{4} } \right] \hfill \\ \end{aligned}$$

where the first species *HCO*_3_^−^ represents the dominating contribution to seawater alkalinity. It is related with the seawater buffer capacity directly.

As shown in Fig. 5, the initial pH value of NaClO_2_ freshwater is about 10.5 and it drops sharply with the addition of HCl. An addition of 6.6×10^−2^ mmol HCl can make the pH value of 0.2 L NaClO_2_ freshwater drop below 7. Whereas the initial pH value of NaClO_2_ seawater is 8.6, which is just a little higher than that of seawater alone. With the addition of HCl, the pH value of NaClO_2_ seawater drops slowly. It requires 1.14×10^−1^ mmol HCl to make the solution pH drop to 7. The result shows that, for NaClO_2_ solution, seawater has a much better buffering ability in maintaining the solution pH than freshwater. It is well known that the buffering ability of seawater is mainly related with the bicarbonate salt which is only 0.48 mmol in 0.2 L seawater. Though the amount of HCl addition, that can make pH value of NaClO_2_ seawater drop to below 7, seems to be much less compared with the molar quantities of bicarbonate salt and NaClO_2_ (5 mmol) in the solution, the buffering ability of seawater might be high enough to enhance the breakthrough time for NaClO_2_ seawater during the cyclic scrubbing experiments.

**Fig. 6:**
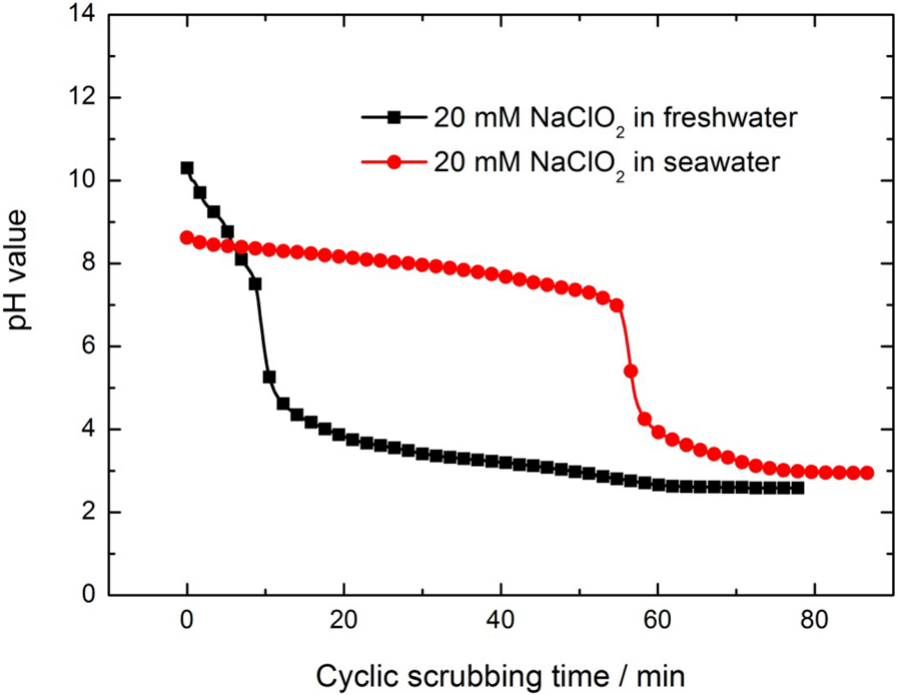
The change in solution pH value during the scrubbing process

The changes of the solution pH during the cyclic scrubbing process are presented in Fig. 6. For 20 mM NaClO_2_ freshwater solution, with the cyclic absorption of NO, the solution pH drops quickly to below 4, resulting that the majority of the NO removal process is in acidic condition. After scrubbing for several minutes, a greenish yellow color is observed in NaClO_2_ freshwater solution, which is confirmed to be ClO_2_. ClO_2_ is formed by the acidic decomposition of NaClO_2_ in Eq. (7) (Yang and Shaw 1998)

7 $$5NaClO_{2} + 4HCl \to 4ClO_{2} + 5NaCl + 2H_{2} O$$

ClO_2_ is found to be the active intermediate for NO oxidation. It does not react with water or ionize in solution, so it could remain as a dissolved gas or be stripped out from the liquid. ClO_2_ escaped from the liquid contributes to the oxidation of NO to NO_2_ in the gas phase

8 $$5NO + 3ClO_{2} + 4H_{2} O \to 5HNO_{3} + 3HCl$$

when the amount of ClO_2_ is slightly in excess of that needed for the NO oxidation, NO will be absorbed completely during the breakthrough time. However, if the amount of ClO_2_ is largely in excess of that needed for the oxidation of NO, the redundant ClO_2_ will escape from the scrubber reactor without making contribution to the oxidation of NO. One can deduce from Figs. 2, 6 that, for 20 mM NaClO_2_ freshwater solution, a certain amount of ClO_2_ has possibly escaped into the atmosphere due to the low pH value during the majority process of NO absorption. In addition, it is reported that, in acidic medium (pH range of 3.5–4.0), a reaction will occur in Eq. (9) (Brogren et al. 1998).

9 $$4ClO_{2}^{ - } + 2H^{ + } \to 2ClO_{2} + ClO_{3}^{ - } + H_{2} O + Cl^{ - }$$

The formation of *ClO*_3_^−^ consumes a small quantity of *ClO*_2_^−^, which makes no contribution to the oxidation of NO, too.

It can be seen from Fig. 6 that, for NaClO_2_ seawater, the solution pH remains above 7 during the major part of the cyclic scrubbing process owning to the buffering ability of seawater. The neutral or slightly alkaline solution is beneficial to suppress the formation of ClO_2_ effectively. In alkaline medium, NO is directly oxidized by *ClO*_2_^−^ in Eq. (1) and NO_2_ is absorbed in Eq. (10).

10 $$4NO_{2} + ClO_{2}^{ - } + 4OH^{ - } \to 4NO_{3}^{ - } + Cl^{ - } + 2H_{2} O$$

Thus, the reason for the extension of the breakthrough time for NaClO_2_ seawater might be mainly ascribed to the improved utilization of NaClO_2_. The acidic decomposition of NaClO_2_ for NaClO_2_ freshwater and further the escape of ClO_2_ into atmosphere have lowered the utilization of NaClO_2_ in freshwater scrubbing solution to some extent. The other factors (such as the transition metal ions, ion strength and solution pH etc.) may also influence the NO absorption by NaClO_2_ seawater, which will be explored in the future in detail and the results will be reported in other forms.

**Fig. 7:**
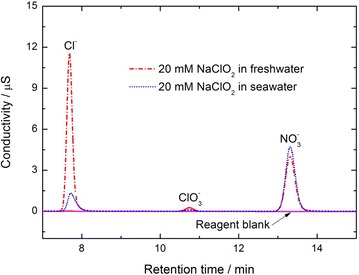
Ion chromatograms of absorption solution

To confirm the composition of reaction products, the anions in the spent liquor are determined using ion chromatography. Ion chromatographic analysis of spent scrubbing liquor and reagent blank is depicted in Fig. 7. Since the concentration of *Cl*^−^ in seawater is relatively too high, it results in that ion chromatography can not be used to analyze the spent liquor of NaClO_2_ seawater scrubbing solution directly. Therefore, the spent liquor of NaClO_2_ seawater scrubbing solution should be treated before doing ion chromatography analysis. Firstly, the spent liquor is diluted 100 times in order to reduce the consumption of Ag cartridge. Then, the diluted solution is filtered by Ag cartridge to remove excessive Cl^−^ ions from the seawater solution. Actually, the spent liquor of NaClO_2_ freshwater scrubbing solution can be analyzed by ion chromatography directly. For comparison, the spent liquor of NaClO_2_ freshwater is also treated by diluting 100 times, but it is not filtered by any Ag cartridge.

Figure 7 indicates that *Cl*^−^ and *NO*_3_^−^ are the major anion products in the spent liquor. There is no *ClO*_2_^−^ in the spent liquor because NaClO_2_ in the solution has been consumed completely during the cyclic scrubbing process. For 20 mM NaClO_2_ freshwater, there is certain amount of *ClO*_3_^−^ in the spent solution, which may be formed by the acidic decomposition of *ClO*_2_^−^ in Eq. (9).

As shown in Fig. 7, the concentration of *Cl*^−^ in freshwater represents the actual value of *Cl*^−^ which exist in the spent liquor and comes from the NaClO_2_ oxidant. While the concentration of *Cl*^−^ in seawater represents the value of *Cl*^−^ left in the sample solution after being filtered by Ag cartridge.

Note that the treatment of the spent liquor does not affect the concentrations of other ions, such as *NO*_3_^−^ and *ClO*_3_^−^. As shown in Fig. 7, the concentration of *NO*_3_^−^ in spent liquor of NaClO_2_ seawater is a little higher than that in freshwater. It means that, with the same concentration of NaClO_2_, the seawater solution has absorbed more NO during the cyclic scrubbing process than freshwater solution. It also demonstrates that, seawater is helpful to improve the utilization of NaClO_2_ compared with freshwater.

According to the measurement results of ion chromatography, the NaClO_2_ utilization for the scrubbing solution may be calculated approximately as follows. For 20 mM NaClO_2_ seawater, *NO*_3_^−^ ion concentration in the spent solution is about 2.72 mmol that can be calculated from the measurement result of ion chromatograms. As *NO*_3_^−^ derives from the absorption of NO by NaClO_2_ through the Eq. (11), so one can deduce that, the molar quantity of *NO*_3_^−^ ions correspond to equal molar of NO, which has consumed 2.04 mmol NaClO_2_ during this process.

11 $$4NO + 3NaClO_{2} + 4NaOH \to 4NaNO_{3} + 3NaCl + 2H_{2} O$$

Because NO is absorbed completely during the breakthrough time, the amount of NO can be calculated approximately as the product of the flow rate of flue gas, NO concentration and the breakthrough time, which is about 4.02 mmol. From Eq. (1), we can see that, NO_2_ in the exit flue gas derives from NO oxidation by NaClO_2_, which can be calculated by substracting the molar quantity of 2.72 mmol from the molar quantity of 4.02 mmol. From Eq. (1), it can be seen that 1.3 mmol NO_2_ has consumed 0.65 mmol NaClO_2_. Therefore, the sum of NaClO_2_ that has made contribution to the absorption of NO_x_ during the scrubbing process is 2.69 mmol. Since the addition of NaClO_2_ in the initial seawater solution is 4 mmol, the utilization of NaClO_2_ in seawater solution can be calculated as 67.25 %.

Similarly, for 20 mM NaClO_2_ freshwater, *NO*_3_^−^ ion concentration in the spent liquor is about 2.34 mmol, which has consumed 1.76 mmol NaClO_2_ according to Eq. (12). NO that has been absorbed by NaClO_2_ freshwater during the whole breakthrough time is about 3.29 mmol. NO_2_ in the exit gas is 0.95 mmol, which has consumed 0.48 mmol NaClO_2_ accordingly. Thus the sum of NaClO_2_ that has made contribution to the absorption of NO_x_ during the whole scrubbing process is 2.24 mmol. Since the addition of NaClO_2_ in the initial freshwater solution is also 4 mmol, the utilization of NaClO_2_ in freshwater solution can be calculated as 56 %.

12 $$4NO + 3NaClO_{2} + 2H_{2} O \to 4HNO_{3} + 3NaCl$$

After in all, the utilization of NaClO_2_ in seawater is enhanced by about 20.1 % compared with that of NaClO_2_ in freshwater solution, which agrees well with the comparison result of the breakthrough times in Fig. 3.

## Conclusion

In this work, the effect of seawater on NO removal of NaClO_2_ solution has been investigated in a cyclic scrubbing mode. Compared with NaClO_2_ freshwater solution, NaClO_2_ seawater solution exhibits much longer breakthrough time for NO absorption. The result of blank experiment and acidic titration demonstrates that the extension of breakthrough time for NaClO_2_ seawater might be ascribed to the buffering ability of seawater, which could suppress the acidic decomposition of NaClO_2_ into ClO_2_. The reduction of ClO_2_ escape may be the main reason for the enhancement of NO removal for NaClO_2_ seawater during the cyclic scrubbing process. The analysis of liquid reaction products indicated that NaClO_2_ has been consumed completely during the cyclic scrubbing process and turned into *Cl*^−^ in the spent liquor. The calculation result from the ion chromatography demonstrates the enhancement of NaClO_2_ utilization in seawater, which agrees well with the breakthrough times for NO removal. This work suggests that NaClO_2_ seawater solution is a promising choice for NOx removal, especially for the applications in onshore power plants and ocean-going ships.
